# What Is It?

**Published:** 1908-04

**Authors:** August Wolf

**Affiliations:** Spokane, Washington


### What Is It?

SPOKANE, WASH., February, 1908.

*Editor Texas Medical Journal:*

Dr. W. A. Egbert, a practicing physician in Spokane, has just awakened from a sleep of 312 hours, during which a dozen of the foremost medical men in Eastern Washington failed to arouse him. While the happenings of the thirteen days are as a blank to him, he came out of the sleep refreshed and with a cleared vision and a healthy appetite, declaring also that his heart action is better than it has been in fifteen years. The case is one of the strangest known to the profession in the Northwest.

Dr. Egbert retired at the usual hour the night of January 26th, occupying a couch in a room adjoining his office. He was found on the floor the following morning by Walter H. Thomas, who shares the suite. Failing to arouse the sleeping man, Thomas called Captain John Gray, a member of the city council, who summoned several physicians, among them Drs. D. C. Newman and E. L. Ingersoll, but their efforts were of no avail. Dr. Egbert remained in a comatose condition until the night of February 1st, when he was conveyed to St. Luke's Hospital, where ten physicians worked over him but were unable to awaken him. On the evening of the thirteenth day the physician literally jumped from the bed and asked where he was. He was discharged a few days later.

Speaking of the case, Dr. Egbert said to the correspondent:

"For a few days before starting in on my long sleep, I had not been feeling well, and yet did not call myself sick. I had taken a little strychnia and several doses of aconite for a cold, but I know nothing in the whole curriculum of medicine that would bring on such a condition as that which I have passed through. It was certainly a good long nap and at its close I felt refreshed, and my heart's action is better today than it has been for fifteen years. The whole period is a blank to me. I had no pain, and the doctors tell me there were no tetanus spasms, such as might be produced by too much strychnia. The sleep was not the result of liquor or dope of any kind, as I had taken no narcotic."

None of the physicians in attendance have attempted to diagnose the case, and they declare it the strangest attack that has yet come into their experience. Dr. Newman said that in the long sleep the physician's body was limp and that he appeared like a man dying. This belief was shared by the attendants and nurses, who on the fifth day said the dissolution was only a matter of hours.

August Wolf,
Spokane, Washington.

